# Controlled Ovarian Hyperstimulation with Intrauterine Insemination Is More Successful After r-hCG Administration Than Spontaneous LH Surge

**Published:** 2017

**Authors:** Evan Taerk, Edward Hughes, Cassandra Greenberg, Michael Neal, Shilpa Amin, Mehrnoosh Faghih, Megan Karnis

**Affiliations:** - Division of Gynecologic Reproductive Endocrinology and Infertility, Department of Obstetrics and Gynecology, McMaster University, Hamilton, Ontario, Canada

**Keywords:** Controlled ovarian hyperstimulation, hCG, Infertility, Intrauterine insemination, LH surge, Subfertility

## Abstract

**Background:**

The purpose of this study was to evaluate whether clinical pregnancy rate is affected by timing intrauterine insemination (IUI) according to serum LH surge, r-hCG trigger, or a combination of LH surge and r-hCG trigger in controlled ovarian hyperstimulation (COH) cycles for patients with a variety of infertility etiologies.

**Methods:**

The last 365 consecutive COH-IUI cycles performed at ONE Fertility Burlington in 2014 were reviewed and categorized according to method of IUI timing. Associations between categorical variables were analyzed using a combination of Chi-square and Fisher’s Exact tests, and between continuous variables using independent sample t-tests and logistic regression to a level of significance of p<0.05.

**Results:**

The overall clinical pregnancy rate in this sample was 18.1% (66/365). Administration of r-hCG prior to IUI resulted in a higher clinical pregnancy rate compared with spontaneous serum LH surge: 18.2% *vs*. 5.8%, p=0.012. Patients in whom r-hCG was administered concomitantly with a serum LH surge had a higher clinical pregnancy than the r-hCG trigger group (30.8% *vs*. 18.2%, p=0.004) and LH surge group (30.8% *vs*. 5.8%, p<0.001). A sub-group analysis revealed that patients receiving r-FSH, rather than clomiphene or letrozole, had a significantly higher clinical pregnancy rate after r-hCG trigger as compared to the LH surge group (21.7% *vs*. 2.1%, p=0.01).

**Conclusion:**

In subfertile couples undergoing COH-IUI, r-hCG administration was associated with an increased clinical pregnancy rate compared with spontaneous serum LH surge. When r-hCG was administered concomitantly with a serum LH surge, this benefit was amplified. The effect appears to be of particular importance in r-FSH-medicated cycles.

## Introduction

Controlled ovarian hyperstimulation-intrauterine insemination (COH-IUI) is often the first-line treatment for unexplained and other types of subfertility. The success of COH-IUI depends on a variety of factors including maternal age, duration of infertility, diagnosis, COH protocol, number of previous treatment cycles, semen parameters and preparation technique, and timing of IUI (1). Initial strategies to improve IUI timing relative to ovulation involved the use of urine luteinizing hormone (LH) detection kits (2), which resulted in superior pregnancy rates when compared to more traditional means of IUI timing involving basal body temperature and prior cycle length (3). It remains unclear whether the introduction of human chorionic gonadotropin (r-hCG) as a method of triggering ovulation for IUI timing results in a higher clinical pregnancy rate when compared to IUI timed according to spontaneous LH surge.

Multiple studies have sought to determine which method is superior and have produced conflicting results (4–10). The variability in their results can likely be explained by differences in medication protocols utilized, baseline characteristics of study participants, the method by which an LH surge was detected, and the retrospective nature of these studies. The first randomized trial (RCT) to address this question showed no difference in clinical pregnancy rates between patients with IUI timed according to serum LH surge or r-hCG trigger (8). However, this study only included patients receiving clomiphene citrate (CC) for COH. More recently, an RCT by Kyrou et al. (10) also found no difference in clinical pregnancy rates between patients undergoing IUI by serum LH surge and r-hCG trigger timing, but this study only included unmedicated cycles.

To date, only one study has investigated the optimal method of IUI timing in COH studies using a variety of COH medication protocols including CC, follicle-stimulating hormone (r-FSH), and letrozole (9). The authors demonstrated higher clinical pregnancy rates in medicated IUI cycles in which r-hCG was administered as compared to cycles in which IUI was timed according to serum LH surge. However, this study only included patients with PCOS and unexplained infertility, and was further limited by the fact that most patients undergoing medicated-IUI received r-hCG irrespective of specific cycle characteristics.

The current study evaluated whether IUI timing with serum LH surge, r-hCG trigger, or a combination of serum LH surge and r-hCG trigger during COH-IUI cycles impacts clinical pregnancy rates in patients with a variety of infertility etiologies.

## Methods

The outcomes of the most recent 371 consecutive patients undergoing COH-IUI cycles at ONE Fertility Burlington between August and December 2014 were compared according to method of IUI timing: serum LH surge, administration of r-hCG, or a combination of serum LH surge and r-hCG administration. Although some patients underwent multiple COH-IUI cycles during this period, only the most recent cycle was included in the analysis. Inclusion criteria were subfertility of ≥12 months of timed intercourse without a pregnancy, age<40 years, bilateral tubal patency as demonstrated on sonohysterogram or laparoscopy, and a minimum post-sperm wash total motile sperm count (TMC) of ≥5×10^6^ sperm undergoing a complete COH-IUI cycle. Cycles in which IUI was not performed were excluded from the analysis.

COH protocols included recombinant FSH (Puregon©, Merck, or; Gonal-F©, EMD Serono; r-FSH), clomiphene citrate (CC), and letrozole. COH protocol was determined for each couple based on female patient age, duration of infertility, etiology of infertility, previous treatment and other aspects of their fertility profile. In general, the initial goal for oligo- or anovulatory patients was the development of 1–2 mature follicles using either CC or letrozole, while the goal for ovulatory patients was 2–3 follicles using either CC or r-FSH. r-FSH injections were administered starting on day 3 of the menstrual cycle and continued until ovulation at doses ranging from 33–175 *IU/day* (median 58 *IU/day*). r-FSH doses were adjusted according to follicular development. CC was provided orally from day 3 to day 7 of the menstrual cycle in doses ranging from 25–150 *mg/day* (median 50 *mg/day*). Letrozole was provided orally from day 3 to day 7 of the menstrual cycle in doses ranging from 2.5–7.5 *mg/day* (median 5 *mg/day*).

Patients were monitored with serial transvaginal ultrasound and serum estradiol, LH, and progesterone until the time of either serum LH surge or r-hCG administration. LH-surge was defined as an increase in LH level ≥200% over the mean of the LH levels on the preceding two days (9) and greater than 10 *IU/l*. r-hCG administration in the form of a single, subcutaneous dose of choriogonadotropin alpha (Ovidrel©, EMD Serono) was offered to patients, in general, once a dominant follicle of >17 *mm* was measured, with an appropriate corresponding estradiol level and a minimum endometrial thickness of 7 *mm*. r-hCG was offered to patients immediately following their ultrasound, prior to reviewing their bloodwork for the day, thus a proportion of patients received r-hCG in the presence of an LH surge. IUI was performed between 09:00 and 11:00, approximately 24 *hr* after either observation of a spontaneous serum LH surge or r-hCG administration. Couples were advised to have intercourse the evening of their IUI. No routine luteal phase support was provided.

Sperm preparation was performed using a gradient separation method. IUI was performed by a physician for patients whose partners had post-wash TMC values ≥5×10^6^ sperm on the day of IUI. No couples within our study utilized donor sperm.

A quantitative serum β-hCG was performed two weeks after IUI and, if positive, repeated two days later to confirm an appropriate increase. Clinical pregnancy was confirmed four to five weeks after IUI through observation of fetal cardiac activity on transvaginal ultrasound.

Clinical pregnancy rate, defined as the visualization of a gestational sac with transvaginal ultrasound after 6.5 weeks of gestation, was compared between the three groups: 1) those having a spontaneous serum LH surge, 2) those receiving r-hCG, and; 3) those having a combination of serum LH surge and r-hCG administration prior to IUI. Also, the differences in pregnancy rate between these three groups were examined according to specific COH protocol and additional variables that might impact treatment outcome, such as age, etiology of infertility, treatment protocol, endometrial thickness, post-sperm wash TMC, and the number of mature follicles ≥16 *mm* at the time of ovulation. Descriptive analyses were performed using SPSS software (IBM Corp., Version 22). Associations between categorical variables were analyzed using a combination of Chi-square and Fisher’s Exact tests. Associations between continuous variables were analyzed using a combination of independent sample t-tests and logistic regression. A p-value of 0.05 was considered significant.

Ethics approval was obtained through the Internal Research Ethics Committee at ONE Fertility in Burlington.

## Results

Of the 371 consecutive patients undergoing medicated IUI cycles at ONE Fertility Burlington between August and December 2014, 365 patients satisfied study inclusion criteria. The clinical pregnancy rate for this population was 18.1% (66/365). The rate of spontaneous miscarriage was 13.6% (9/46). No cases of ectopic pregnancy were observed. No cases of multiple pregnancy were observed.

Table 1 describes the baseline characteristics of patients undergoing IUI timed according to spontaneous serum LH surge (LH surge group), r-hCG administration (r-hCG trigger group), and a combination of serum LH surge and r-hCG administration (combined LH surge-hCG trigger group). The three groups were similar in terms of age, treatment protocol, endometrial thickness at the time of IUI, and post-sperm wash TMC. Indications for treatment included unexplained infertility (30.0%), diminished ovarian reserve (22.0%), ovulatory dysfunction (19.0%), mild male factor infertility (9.3%), endometriosis (5.7%), cervical/ tubal pelvic factor (4%), hypothalamic causes (1.0%), and multi-factorial (9.0%).

**Table 1.** Baseline characteristics of study groups, mean±SD (unless otherwise specified)

	**LH group (n=69)**	**hCG group (n=231)**	**Combined group (n=65)**
**Age (years)**	33.1±3.4	33.3±3.4	32.9±3.4
**Treatment**			
**r-FSH**	68.1%	61.9%	69.2%
**Clomiphene citrate**	26.1%	35.1%	29.2%
**Letrozole**	5.8%	3.0%	1.5%
**Endometrial thickness (*mm*)**	10.1±2.7	10.2±2.2	10.8±2.1
**Peak estradiol (*pmol/L*)**	1561.1±785.1	2163.6±1081.0^*^	2205.1±1000.9^*^
**Post wash TMC (×10^6^)**	34.3±34.0	38.7±43.2	35.6±33.1
**No. of follicles ≥16 *mm***	1.1±0.6	1.4±0.6^*^	1.4±0.6^*^

* p<0.001, compared to LH surge group

The r-hCG trigger group and combined LH surge-hCG trigger group both had higher estradiol concentrations (2163.6 *vs*. 1561.1 *pmol/L*, p< 0.001; 2205.1 *vs*. 1561.1 *pmol/L*, p<0.001, respectively) and a higher number of mature follicles (1.4 *vs*. 1.1, p<0.001; 1.4 *vs*. 1.1, p<0.001, respectively) at the time of trigger as compared to the LH surge group (Table 1), but these two variables were not associated with clinical pregnancy rate. No differences in peak estradiol concentration and number of mature follicles were observed between the r-hCG trigger and combined LH surge-hCG trigger groups.

Table 2 describes clinical pregnancy rates per cycle according to study groups across the different COH treatment protocols. The clinical pregnancy rate was significantly higher in the r-hCG trigger group as compared to the LH surge group (18.2% *vs*. 5.8%, p=0.012). In addition, the combined LH surge-hCG trigger group had a higher clinical pregnancy rate compared with the r-hCG trigger group (30.8% *vs*. 18.2%, p=0.004) and the LH surge group (30.8% *vs*. 5.8%, p<0.001).

**Table 2.** Clinical pregnancy rates per cycles according to study groups across different COH protocols, rate (number of pregnancies/total number of cycles)

	**LH group**	**hCG group**	**Combined group**	**All cycles**
**r-FSH**	2.1% (1/47)	21.7% (31/143)	33.3% (15/45)	20.0% (47/235)
**Clomiphene citrate**	11.1% (2/18)	13.6% (11/81)	26.3% (5/19)	15.3% (18/118)
**Letrozole**	25% (1/4)	0% (0/7)	0% (0/1)	8.3% (1/12)
**All medications**	5.8% (4/69)	18.2% (42/231)	30.8% (20/65)	18.1% (66/365)

**Figure 1.**
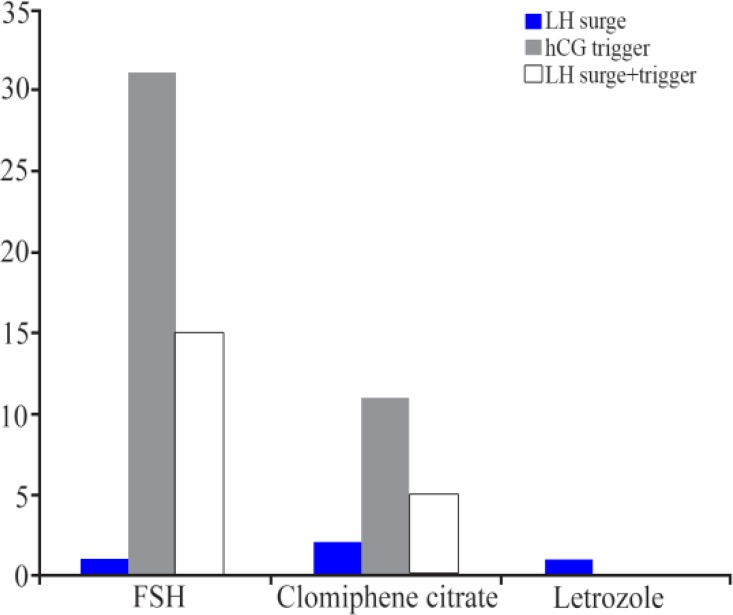
Clinical pregnancy rate according to study groups across different COH protocols

A sub-group analysis according to COH protocol revealed that patients receiving r-FSH had a significantly higher clinical pregnancy rate after r-hCG trigger as compared to the LH surge group (21.7% *vs*. 2.1%, p=0.01) (Figure 1). Those patients receiving r-FSH within the combined LH surge-hCG trigger group also had a higher clinical pregnancy rate when compared to patients in the r-hCG trigger group (33.3% *vs*. 21.7%, p=0.03) and the LH surge group (33.3% *vs*. 2.1%, p< 0.001). No difference in clinical pregnancy rate was observed between the r-hCG trigger group, the LH surge group, or the combined LH surge-hCG trigger group in patients receiving either CC or letrozole.

### Statistical analysis:

Multiple regression analyses were performed investigating the observed association between r-hCG administration and clinical pregnancy rate while controlling for potential variables such as age, diagnosis, COH protocol, peak estradiol concentration, endometrial thickness, post-sperm wash TMC and number of follicles present at the time of IUI. r-hCG administration was observed to be an independent predictor of clinical pregnancy rate (OR 2.34, 95% CI [1.2, 3.3], p=0.05).

## Discussion

This study demonstrated a beneficial effect of r-hCG administration on clinical pregnancy rates in COH-IUI cycles in patients with a variety of infertility etiologies. This effect is amplified when r-hCG is administered in conjunction with a spontaneous surge in serum LH. Although the beneficial effect of r-hCG administration is observed with each treatment protocol, it appears to be of particular value in IUI cycles using r-FSH for ovarian stimulation.

Although no baseline difference was observed between the r-hCG trigger group and the LH surge group in terms of peak estradiol concentration and number of follicles present at ovulation, these variables were not predictive of clinical pregnancy. This finding is not surprising given the tendency to recommend early r-hCG trigger in the presence of a high estradiol value, or a high number of developing follicles, in an effort to minimize the rate of multiple pregnancy.

Among the multitude of factors contributing to IUI success, the appropriate timing of IUI relative to ovulation appears to be imperative (11). Ovulation occurs 24–56 *hr* following the onset of a spontaneous LH surge (12), while ovulation has been observed to occur 36–48 *hr* after r-hCG administration (13). Studies comparing medicated cycles, in which IUI is performed either 24 or 36 *hr* after r-hCG administration, have not demonstrated a difference in clinical pregnancy rate (14).

Studies investigating the optimal method of IUI timing relative to ovulation have produced mixed results. The first RCT to address this question showed no difference in clinical pregnancy rates between patients with IUI timed according to serum LH surge *vs*. r-hCG trigger (8). However, this study only included patients receiving CC for COH. Given the range of medications commonly employed in IUI cycles, and their varying effects on follicular development and endometrial receptivity (15, 16), these findings may not be generalizable to other treatment groups. The current study includes a variety of COH medication protocols to account for these potential differences. In addition, pregnancy rates in that study were extremely low (∼4%) compared with the current study (15.3%), which is consistent with published rates of IUI success (CARTR, 2014).

A recent RCT by Kyrou et al. (10) also found no difference in clinical pregnancy rates between patients undergoing IUI timed by serum LH surge and r-hCG trigger. This study, however, only included unmedicated cycles. Ample evidence exists to suggest that the timing and physiology of ovulation during medicated cycles differs significantly from unmedicated cycles and thus methods of timing IUI likely differ significantly as well (16, 17). Again, the current study attempted to address these differences in physiology by including a variety of COH medication protocols. As well, the majority of cycles in the Kyrou et al. (10) study involved same-sex female couples undergoing IUI with donor sperm so, again, conclusions from this study may not be generalizable to subfertile groups.

A meta-analysis by Cantineau et al. (14) included four studies comparing IUI cycles timed according to a spontaneous LH surge with those IUI cycles timed according to r-hCG administration. They found no difference in clinical pregnancy rates between these two groups. However, three of the four included studies used urine LH kits rather than serum measurements to determine LH surge. Urine LH predictor kits are associated with a high rate of false negative results (∼35%) due to their inability to detect surges below 40 *IU/L* (18), and some women may ovulate before LH can be detected in the urine (19). The fourth included study, which did measure serum LH, was the unmedicated-IUI trial performed by Kyrou et al. (10).

In a retrospective study of infertile patients undergoing medicated-IUI, Mitwally et al. (9) observed a beneficial effect of r-hCG administration on clinical pregnancy rate. However, only patients with PCOS and unexplained infertility were included and r-hCG was administered in almost all r-FSH cycles regardless of cycles characteristics again raising the issue of generalizability.

The observation of a beneficial effect of r-hCG administration might reflect the challenge of defining an LH surge, especially when a surge can last up to 48 *hr*, and thus timing an IUI according to r-hCG administration, rather than an LH surge, may be a more effective method of achieving pregnancy (20). Similar to the Mitwally et al.’s study (9), the current study also observed a beneficial effect of r-hCG administration in cycles using r-FSH. r-FSH tends to produce a rapid rise in serum estradiol and the tendency towards a premature and inadequate LH surge, which appears to be mitigated by the use of an r-hCG trigger (21).

Our finding of a higher pregnancy rate in cycles in which r-hCG was administered in the presence of an LH surge has also been observed in a previous study (9) and may reflect the fact that timing IUI exclusively according to r-hCG administration may occasionally result in the triggering of immature follicles and reduced pregnancy rates (6).

Contrary to Mitwally et al.’s study (9) that observed a beneficial effect of LH surge over rhCG administration in CC cycles, no significant difference between the two methods of IUI timing was observed in this study when our analysis was restricted to CC cycles. Although the small number of CC cycles included in this analysis limit our ability to adequately address this issue, the timing of IUI relative to ovulation may differ in CC cycles as compared to r-FSH cycles given the need of hypothalamus to overcome the estrogen-receptor antagonism produced by CC (8). This does not, however, account for the challenge of defining an LH surge and timing IUI, accordingly.

Of the 300 cycles reviewed in the current study, 11 utilized letrozole resulting in one pregnancy. It is thus unreasonable to draw conclusions regarding the optimal means of timing IUI with letrozole treatment based on this study.

Although the current study includes an accurate method of LH surge detection, a diverse population of patients and diagnoses, and a variety of COH protocols, several limitations must be noted. Firstly, the retrospective design introduces the possibility of both selection and information biases. Secondly, the lack of standardized criteria for triggering ovulation limits the generalizability of these results. Lastly, although the absence of multiple pregnancy within the study group likely reflects the conservative approach to COH practiced at our centre rather than an inherent difference in our patient population, given the well-established risk of multiple pregnancy reported in previous COH-IUI studies (14), this observation may also limit the generalizability of our results. A larger randomized trial employing similarly broad inclusion criteria and methods would further help to elucidate this important research question.

## Conclusion

r-hCG administration as a means of timing IUI during medicated cycles appears to improve clinical pregnancy rates when compared to the observation of a spontaneous LH surge. Furthermore, when r-hCG is administered concomitantly with a serum LH surge, this benefit appears to be amplified. This effect appears to be of particular importance in r-FSH-medicated cycles.
